# Heat drastically alters floral color and pigment composition without affecting flower conspicuousness

**DOI:** 10.1002/ajb2.70096

**Published:** 2025-09-09

**Authors:** Eduardo Narbona, Francisco Perfectti, Adela González‐Megías, Luis Navarro, José C. del Valle, Cristina Armas, José M. Gómez

**Affiliations:** ^1^ Dpto. de Biología Molecular e Ingeniería Bioquímica Universidad Pablo de Olavide Sevilla Spain; ^2^ Dpto. de Genética Universidad de Granada Granada Spain; ^3^ Research Unit Modeling Nature Universidad de Granada Granada; ^4^ Dpto. de Zoología Universidad de Granada Granada Spain; ^5^ Dpto. de Biología Vegetal y Ciencias del Suelo Universidad de Vigo Vigo Spain; ^6^ Dpto. de Botánica y Ecología Vegetal Universidad de Sevilla Sevilla Spain; ^7^ Estación Experimental de Zonas Áridas (EEZA‐CSIC) Almería Spain

**Keywords:** Brassicaceae, chromatic contrast, flower color, high temperature, *Moricandia arvensis*, phenotypic plasticity, plant–pollinator interactions, reflectance spectra, UV‐absorbing pigments, visual modelling

## Abstract

**Premise:**

Floral pigments primarily serve to attract pollinators through color display and also contribute to protection against environmental stress. Although pigment composition can be plastically altered under stress, its impact on pollinator color perception remains poorly understood. *Moricandia arvensis* (Brassicaceae) exhibits seasonal floral dimorphism, with lilac spring flowers and white summer flowers. This study examines how heat‐driven shifts in floral pigments alter flower color and its perception by pollinators.

**Methods:**

We conducted a comparative analysis of spring and summer floral morphs in a natural population by measuring petal spectral reflectance, analyzing absorption spectra of petal extracts, and modeling floral color in the visual systems of major pollinator functional groups. Additionally, UHPLC‐ESI‐MS/MS analysis was conducted under controlled conditions to characterize differences in phenolic profiles.

**Results:**

Spring flowers exhibited strong UV reflectance and a reduction in reflectance in the green spectrum, whereas summer flowers showed no UV reflectance and high reflectance in the visible range. Anthocyanins were detected only in spring flowers, while summer flowers accumulated high levels of UV‐absorbing flavonoids. Despite these differences, both floral morphs remained visually conspicuous to hymenopterans, dipterans, lepidopterans, and coleopterans. Summer flowers produced twice as many phenolic compounds and accumulated higher concentrations, with ferulic acid and kaempferol derivatives the most prominent.

**Conclusions:**

White summer flowers of *Moricandia arvensis* are not merely anthocyanin‐deficient but exhibit a distinct profile of UV‐absorbing phenolics that may confer heat tolerance while preserving floral conspicuousness to pollinators. These findings highlight the role of multifunctional traits in the evolution of flower color.

Flowers consist of a complex suite of traits (Khojayori et al., 2024), each exposed to multiple selection pressures from both biotic and abiotic factors (Strauss and Whittall, 2006; Rusman et al., 2019; Briggs and Anderson, 2025). In animal‐pollinated angiosperms, flower color is a key trait that acts as a visual cue to attract pollinators (van der Kooi et al., 2019), ultimately influencing reproductive success (Caruso et al., 2019). Flower color primarily results from pigment accumulation, and owing to their biochemical properties, these pigments may potentially play other physiological roles in flowers (Narbona et al., 2025). The major pigment groups (i.e. flavonoids, carotenoids, chlorophylls and betalains) can mitigate oxidative stress and may be implicated in various responses to environmental stressors, including extreme temperatures, solar radiation, drought, and pathogens (Grotewold, 2006; Davies et al., 2022; Narbona et al., 2025).

The most frequent group of pigments in flowers—and the most diverse in terms of compounds and functions—are the flavonoids (Schwinn and Davies, 2004; Iwashina, 2015; Wen et al., 2020). Anthocyanins, a subgroup of flavonoids, are responsible for most pink, blue, violet, red, and orange colorations in flowers (Tanaka et al., 2008), and are particularly important for attracting pollinators (Narbona et al., 2021). Although anthocyanins can exhibit high antioxidant capacity, their protective role has been investigated primarily in vegetative tissues (Landi et al., 2015; Naing and Kim, 2021). It has been suggested that the presence and concentration of floral anthocyanins may be related to inhabiting colder regions, although the underlying mechanisms remain unclear (Dalrymple et al., 2020; Dellinger et al., 2025, in this issue). Recently, it has been shown that other flavonoid groups, such as flavonols and flavones, are ubiquitous in flowers (Narbona et al., 2025). These compounds are imperceptible to humans and strongly absorb ultraviolet (UV) light, hence they are commonly referred to as UV‐absorbing flavonoids. They also play an important role in pollinator attraction by forming UV bullseye patterns or nectar guides (Harborne and Smith, 1978; Sasaki and Takahashi, 2002). In contrast to anthocyanins, increasing evidence suggests the protective role of UV‐absorbing flavonoids against various types of stress in flowers. For instance, using transgenic plants of *Arabidopsis thaliana*, it has been demonstrated that the production of phenylacylated‐flavonols in flowers, including petals, contributed to UV‐B response (Tohge et al., 2016). Additionally, *A. thaliana* plants exposed to relatively high temperature conditions (35°C for 5 h) exhibited changes in the concentrations of several flavonol derivatives present in buds and flowers (Borghi et al., 2019). Furthermore, increased flavonol content in petals has been shown to indirectly enhance pollen viability (Koski and Ashman, 2015; Koski et al., 2022), pollen tube growth and seed set (Chen et al., 2021), as well as resistance to desiccation (Todesco et al., 2022). Finally, both flavonols and flavones have been shown to exhibit activity against a variety of herbivores and pathogens, including fungi, bacteria or viruses (Gronquist et al., 2001; Zhang et al., 2013; Górniak et al., 2019; Kumar et al., 2023).

Although a growing body of evidence supports that the production of UV‐absorbing flavonoids in floral tissues is influenced by environmental factors, the extent to which pollinators perceive and respond to these pigment variations remains poorly understood. Most major pollinator groups—including hymenopterans, dipterans, lepidopterans, coleopterans, and birds—exhibit pronounced sensitivity to UV light (Dyer et al., 2021; Narbona et al., 2021). Accordingly, variation in the concentration or spatial distribution of UV‐absorbing flavonoids within floral tissues is expected to modulate pollinator behavior. This is illustrated in *Petunia exserta*, where hawkmoths preferentially visit flowers with higher flavonol concentrations (Sheehan et al., 2016). Conversely, bees and hoverflies do not show consistent preferences for UV‐absorbing versus UV‐reflecting floral colors (Papiorek et al., 2016; An et al., 2018), which are mostly linked to the accumulation of UV‐absorbing flavonoids (Narbona et al., 2021; 2025). Such contrasting responses suggest that the presence of UV‐absorbing flavonoids does not exert a universally positive or negative influence on pollinator attraction (e.g., Koski and Ashman, 2014; DeMarche et al., 2015; Brock et al., 2016; Todesco et al., 2022). This variability may stem from a limited understanding of how pollinators perceive the color shifts induced by the accumulation of UV‐absorbing flavonoids in flowers.

In this study, we investigated how heat‐induced changes in floral flavonoid pigments—specifically both anthocyanins and UV‐absorbing flavonols—affect flower color and how these changes influence perception by pollinators. We focused on *Moricandia arvensis* (L.) DC. (Brassicaceae), an annual to short‐lived perennial species in which individual plants produce two distinct floral morphs due to within individual phenotypic plasticity in response to abiotic conditions (Gómez et al., 2020, 2022, 2025a). Over the flowering season, this species produces large, cross‐shaped lilac flowers in spring, while in summer, the same individuals develop smaller, rounded white flowers. This floral polyphenism occurs across its entire distribution in the drylands of the Western Mediterranean Basin (Gómez et al., 2022, 2024). Lilac spring flowers predominantly accumulate cyanidin derivatives, whereas white summer flowers lack anthocyanins and instead accumulate flavonol derivatives (Gómez et al., 2020). Experimental studies showed that this flower color shift is highly plastic and is primary triggered by summer‐like temperatures (30ºC and 35ºC; Gómez et al., 2020). Flowers of *M. arvensis* attract a diverse array of insects, with large long‐tongued bees being the primary pollinator group for spring flowers; in contrast, summer flowers are primarily visited by small short‐tongued bees, large long‐tongued bees, large butterflies, beeflies, and beetles (Gómez et al., 2022, 2024, 2025a). Therefore, *M. arvensis* represents an ideal study system to investigate how temperature‐driven changes in floral pigment composition influence flower color as perceived by pollinators. This approach could help determine whether abiotic factors enhance or constrain pollinator interactions, providing insights into how biological and climatic factors interact to shape floral evolution (Traine et al., 2024). We investigated how variations in floral flavonoid composition influence flower color and how these changes are perceived by different pollinator groups. To achieve this, we conducted a comparative analysis of the spring and summer floral morphs in a natural population. Specifically, we: (1) characterized floral color by measuring petal spectral reflectance; (2) estimated floral pigment composition through absorption spectra of petal extracts; (3) modeled floral color perception using the visual systems of representative species from Hymenoptera, Diptera, Lepidoptera, and Coleoptera, incorporating both spring and summer background environments; and (4) generated two simulated reflectance spectra—one representing a summer flower lacking flavonols and another a spring flower with added flavonols—and calculated their conspicuousness. In addition, UHPLC‐ESI‐MS/MS analysis was performed to characterize differences in flavonoid and other phenolic compound composition between spring and summer flowers grown under controlled conditions.

## MATERIALS AND METHODS

### Sampling

Data were obtained during a field study conducted in the Negratín population (37° 33.7′ N, 3° 57.5′ W; Granada province, Spain). In February 2019, we marked 100 plants at the onset of the flowering period. In each plant, one flower was collected during both the spring and summer flowering peaks (March and July, respectively). The petals of each flower were immediately dissected, with one petal used for reflectance spectra measurements (flower color quantification study) and two petals used for pigment extraction (flower pigment composition study). Flower dissection and subsequent measurements were performed *in situ* using a mobile laboratory installed in a motorhome.

### Flower color quantification and conspicuousness to pollinators

UV–Vis spectral reflectance was measured using a FLAME spectrophotometer (Ocean Insight Inc., Orlando, Florida, USA) equipped with a deuterium–tungsten light source (200–2000 nm) and a probe holder to make measurements at 45° angle from the sample. The spectrophotometer was calibrated before each measurement using a WS‐1‐SL white standard and the Ocean View 2.0 software. We set an integration time of 2 s and a boxcar width of 12 to maximize light collection during reflectance measurements and to minimize sporadic, erratic reflectance values at individual wavelengths (White et al., 2015). We processed the spectral data within the 300–700 nm range, corresponding to the wavelengths most relevant to pollinator visual systems (White et al., 2015; Narbona et al., 2021), using the ‘prospec’ function from the R‐package ‘pavo’ and applying a smoothness parameter of 0.20 (Maia et al., 2019). For graphical purposes, we used the ‘aggplot’ function to aggregate the spectra from the same sampling period (i.e., spring and summer) and subsequently calculated the average and standard error (S.E.) values.

The flower reflectance data was modelled in the visual systems of the main functional groups of insects that visit both the spring and summer flowers of *M. arvensis*: long‐ and short‐tongued bees, large butterflies, large beeflies, and beetles (Gómez et al., 2020, 2024, 2025a). We used the trichromatic vision model of *Apis mellifera* (Apidae) for the four groups of bees, the tetrachromatic visual model of *Papilio xuthus* (Papilionidae) for butterflies, the tetrachromatic model of *Eristalis tenax* (Syrphidae) for beeflies, and the trichromatic visual system of *Pygopleurus israelitus* (Glaphyridae) for beetles (Chittka, 1992; Koshitaka et al., 2008; Martínez‐Harms et al., 2012; An et al., 2018). The visual systems were modelled using the R­package ‘pavo’ (Maia et al., 2019), with D65 as standard illuminant and von Kries' chromatic adaptation transformation, as detailed in similar studies (León‐Osper and Narbona 2022; del Valle et al., 2025). Additionally, for the specific case of bees we considered a hyperbolic transformed quantum catch. In spring, plants grew in an environment dominated by herbaceous species; thus, we used the mean of 230 green leaves background as described by Chittka et al., (1994). In summer, with virtually no surrounding vegetation, we considered the bare soil as the background, using the average reflectance from 10 soil samples. Among the vision models proposed to represent the color vision of insects (Kelber et al., 2003; Renoult et al., 2017), we chose the color hexagon model for bees (Chittka, 1992), the categorical space model for flies (Troje, 1993), the tetrahedral color space for butterflies (Endler and Mielke, 2005), and the Maxwell triangle for beetles (Maxwell, 1860; Martínez‐Harms et al., 2020).

Differences in color perception of spring and summer flowers by the main groups of pollinators were analyzed using hue, as well as chromatic and achromatic contrast. Hue, the categorized perceptual dimension of color stimuli (Kemp et al., 2015), depends on the specific color vision model of each pollinator group (Renoult et al., 2017). We analyzed flower color conspicuousness using chromatic contrast, which plays a predominant role in stimulus detection by bees and other pollinators, such as flies, beetles, and butterflies (Giurfa et al., 1996; Koshitaka et al., 2008; Martínez‐Harms et al., 2012; García et al., 2021). Chromatic contrast was calculated using the ‘coldist’ function from the R‐package ‘pavo’ (Maia et al., 2019), which measures Euclidean distance units (EU) between the flower's color loci and the achromatic center, based on the spectral sensitivities of photoreceptors in all color space models (van der Kooi and Kelber, 2022; van der Kooi and Spaethe, 2025). For the hymenopteran visual model, we considered ≥ 0.11 EU as the discriminability threshold (Dyer et al., 2012). This threshold has been demonstrated to reliably distinguish between two color loci in the hexagon model (or between the color loci and the background), even under absolute conditioning that better simulates natural conditions (Giurfa, 2004; Dyer et al., 2012). It is important to note that this threshold is conservative, as bumblebees have demonstrated the ability to discriminate floral colors at distances as small as 0.06 EU (Dyer and Chittka, 2004). In addition, flower detection at longer distances by bees primarily relies on achromatic contrast, which pertains to the difference in excitation of the green receptor between a stimulus and its background (Giurfa et al., 1996; Dyer et al., 2008). Achromatic contrast for bees was calculated as the absolute difference between the green receptor excitation and 0.5, which represents the background excitation level (Spaethe et al., 2001).

### Reflectance spectra simulations and spontaneous anthocyanin‐lacking mutants

To disentangle the role of flavonols in the conspicuousness of *M. arvensis* flowers, we generated two simulated reflectance spectra using the mean reflectance values of spring and summer flowers from the Negratín population, as calculated in the previous section. To simulate a summer flower lacking flavonols, we combined the UV range (300–400 nm) of spring flowers with the visible range (400–700 nm) of summer flowers. Conversely, to simulate a spring flower with flavonols, we combined the UV range of summer flowers with the visible range of spring flowers. Because the two flower types differed in maximum reflectance (Appendix S1), their mean reflectance values were manually adjusted to ensure continuity between spectra. For simulated spring flowers with added flavonols, the UV portion of the reflectance curve was shifted toward longer wavelengths to simulate the effect of UV‐absorbing flavonols. Conversely, for simulated summer flowers lacking flavonols, the reflectance spectrum was adjusted toward shorter wavelengths to allow reflectance in the UV range, thereby representing the absence of flavonols.

In spring 2020, three spontaneous mutants completely lacking anthocyanins throughout the plant were identified in a natural population of *M. arvensis* in La Malahá (37° 08.4' N, 3° 43.9' W; Granada province, Spain). Plants in this population exhibit similar floral phenotypic plasticity—namely, comparable morphological differences between spring and summer flowers—as previously reported in the Negratín population (Gómez et al., 2020, 2022). These anthocyanin‐deficient mutants lack pigmentation in both vegetative and reproductive tissues, a rare condition in wild populations (<1%). However, such individuals may occasionally arise in very large populations (e.g., Coburn et al., 2015; Twyford et al., 2018; del Valle et al., 2019). The mutant individuals displayed flowers with the typical spring morphology (i.e., large and cross‐shaped flowers), yet their petals were completely white. One flower per plant was collected, and its reflectance spectrum was measured following the methodology described above. Subsequently, the mean reflectance spectra of the three mutant individuals, along with the simulated reflectance spectra, were modeled within the bee visual system to evaluate their chromatic conspicuousness.

### Flower pigment composition

To assess how flower color is influenced by pigment composition, we conducted an absorbance analysis of petal extracts. Petals were submerged in 1.5 mL methanol:HCl (99:1 v:v) within a 2 mL screw‐cap O‐ring tube. Pigments were extracted at 4°C in the dark for 24 hours until transfer to long‐term storage at −20°C. We take a photo of the corolla to measure area and estimate petal weight as described in Gómez et al. (2020). Petal homogenization was not performed, as the thinness of the petal tissue was sufficient to extract soluble flavonoids (del Valle et al., 2018).

Spectrophotometric measurements were performed 1–2 months later, using two replicates of 200 µL per sample. Absorbance was estimated as optical density (OD) from 260 to 700 nm with 1 nm steps at 22°C constant temperature in a Multiskan GO microplate spectrophotometer (Thermo Fisher Scientific Inc., Waltham, Massachusetts, USA). We analyzed absorbance spectral data within the 300–700 nm range to ensure comparability with the reflectance measurements. UV‐absorbing flavonoids exhibited characteristic absorption in the UV region of the light spectrum (300–400 nm), whereas anthocyanins showed peak absorbance primarily in the green region, around 500 nm (Narbona et al., 2025). Total UV‐absorbing flavonoids and anthocyanins were quantified based on absorbance at 350 nm and 520 nm, using calibration curves previously calculated of cyanidin‐3‐glucoside chloride and kaempferol‐3‐glucoside, respectively (Gómez et al., 2020). Results were expressed as kaempferol‐3‐glucoside and cyanidin‐3‐glucoside and equivalents per gram of fresh weight (mg g⁻¹ FW).

### Determination of phenolic compounds by UHPLC‐HRMS/MS analyses

We conducted a study under controlled conditions to determine the composition of flavonoids and other phenolic compounds in spring and summer flowers. In spring 2018, seeds were collected from the Malahá population and were germinated in summer 2018; and the resulting seedlings were transplanted into individual plots. Fifteen plants were grown under controlled conditions in growth chambers and subjected to two consecutive treatments. The first treatment simulated spring conditions typical of Mediterranean Spain, with a temperature of 20/10°C (day/night = 10/14 h). Subsequently, the plants were exposed to conditions simulating a hot Mediterranean summer with a temperature of 35/25°C (day/night = 16/8 h (see Gómez et al., 2020). Experimental studies that independently manipulated temperature and photoperiod demonstrate that temperature alone is sufficient to induce the change in flower color (Perfectti F., unpublished results). In seven plants, two petals of one flower were collected from each treatment (spring and summer), and pigments were extracted using acidified methanol, following the previously described protocol. Samples were stored at −20°C until further pigment identification and quantification.

UV‐absorbing flavonoids and other classes of phenolic compounds (i.e. coumarins, phenolic aldehydes, hydroxycinnamic acids, benzoic acids, and hydroxybenzoic acids) were analyzed with an Ultra High Pressure Liquid Chromatography (UHPLC) system consisting of a quaternary UHPLC Dionex Ultimate 3000 SD connected to a quadrupole‐orbitrap QExactive hybrid mass spectrometer (MS) (ThermoFisher Scientific, San Jose, California, USA) with a heated electrospray ionization probe (HESI‐II). Xcalibur 4.0 software was used for instrument control and data acquisition. Separation was carried out using an Acquity BEH C18 column (1.7 µm particle size, 100 × 2.1 mm) (Waters) at 40°C at a flow rate of 0.5 ml/min. A binary gradient consisting of (A) water and (B) methanol both containing 0.1% formic acid was used with the following elution profile: 5% B (1 min), linear gradient to 100% B (9 min), 100% B (2 min) and finally 5% B (3 min). The injection volume was 5 µL.

A data dependent acquisition method (Top5) was used in negative mode at resolution 70000 and 17500 at m/z 200 FWHM for Full Scan and Product Ion Scan, respectively. HESI source parameters were: spray voltage, −3.0 kV; S‐lens RF level, 50; capillary temperature, 320°C; sheath and auxiliary gas flow, 60 and 25 respectively (arbitrary units); and probe heater temperature, 400°C. Trace Finder 5.1 software was used for data treatment. The identification was made by comparing the exact masses of the pseudomolecular ion (maximum deviation of 5 ppm) and their fragment ions with the data contained in a database with 127 possible phenolic compounds. The retention time of standard compounds (delta > 0.002) and isotopic pattern scores higher than 80% were also required. Anthocyanins were excluded from our analysis, as they were previously identified through UPLC–ESI–TOF–MS and consist of a homogeneous group of cyanidin‐3‐glucoside derivatives (Gómez et al., 2020).

### Statistical analysis

Because we only sampled two flowers per individual, one per season, we were unable to use the analytical tools designed to evaluate between‐individuals differences in the slope of reaction norms (G × E interactions, Gómez et al., 2025b). So, between‐season comparisons conspicuousness (i.e., achromatic and chromatic contrasts) were performed using random‐intercepts linear mixed model analyses, including season as fixed factor and individual as random factor. These models were implemented with the ‘lmer’ function from the R package ‘lme4’ (Bates et al., 2015). Achromatic and chromatic contrasts of flowers against green foliage and bare soil backgrounds were compared using the Wilcoxon signed‐rank test for paired samples. Between‐season comparisons in the concentrations of individual phenolic compounds obtained in UHPLC analyses were assessed using paired t‐tests (Crawley, 2005). These concentrations were previously normalized to account for variation in relative signal intensity (Shurubor et al., 2005). All statistical analyses were performed using R v.4.4.3 (R Core Team, 2025) and RStudio v.2022.12.0 (RStudio Team, 2022).

## RESULTS

### Flower color and conspicuousness to pollinators

**Figure 1:**
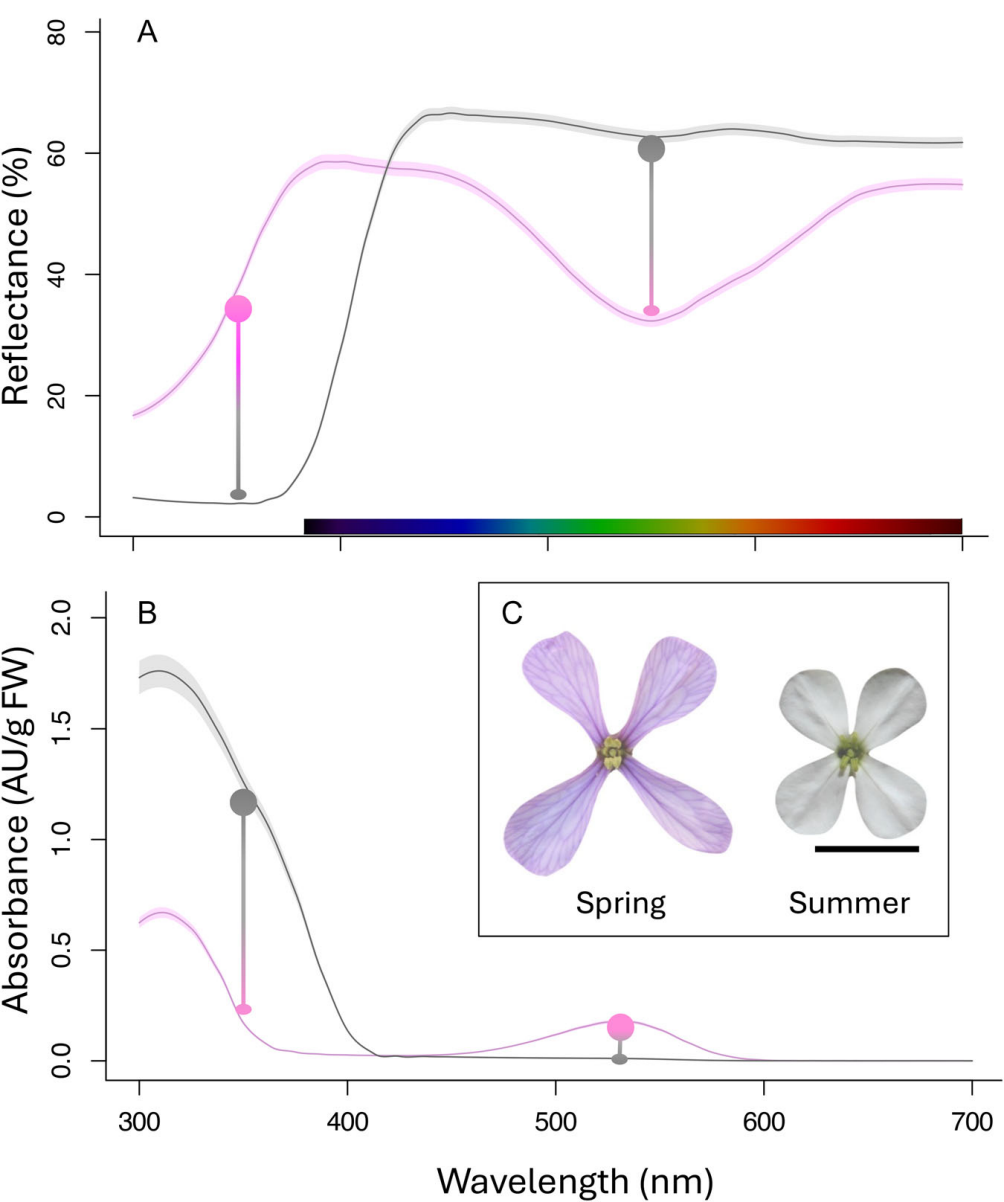
Color features of spring and summer flowers of *Moricandia arvensis*. (A) UV–Vis spectral reflectance (mean ± S.E.) of petals from spring (lilac line) and summer (grey line) flowers. (B) Mass corrected absorbance of acidified methanol extracts of petals from spring (lilac line) and summer (grey line) flowers. (C) Phenotypic characteristics of spring and summer flowers from the same individual plant; scale bar = 10 mm. In panels A and B, the magnitude of changes in the UV (∼350 nm) and green (∼530 nm) regions of the light spectrum are depicted by circles connected by a horizontal line.

Spring (lilac, as perceived by the human eye) and summer (white) flowers exhibited distinct UV‐visible reflectance spectra (Figure 1; Appendix S1). Summer flowers showed no reflectance in the UV region (300–400 nm), whereas spring flowers exhibited reflectance throughout this range, with particularly high values from approximately 350 nm onward. In the visible spectrum, summer flowers exhibited relatively high constant reflectance, whereas spring flowers displayed an absorbance valley around 550 nm.

**Figure 2:**
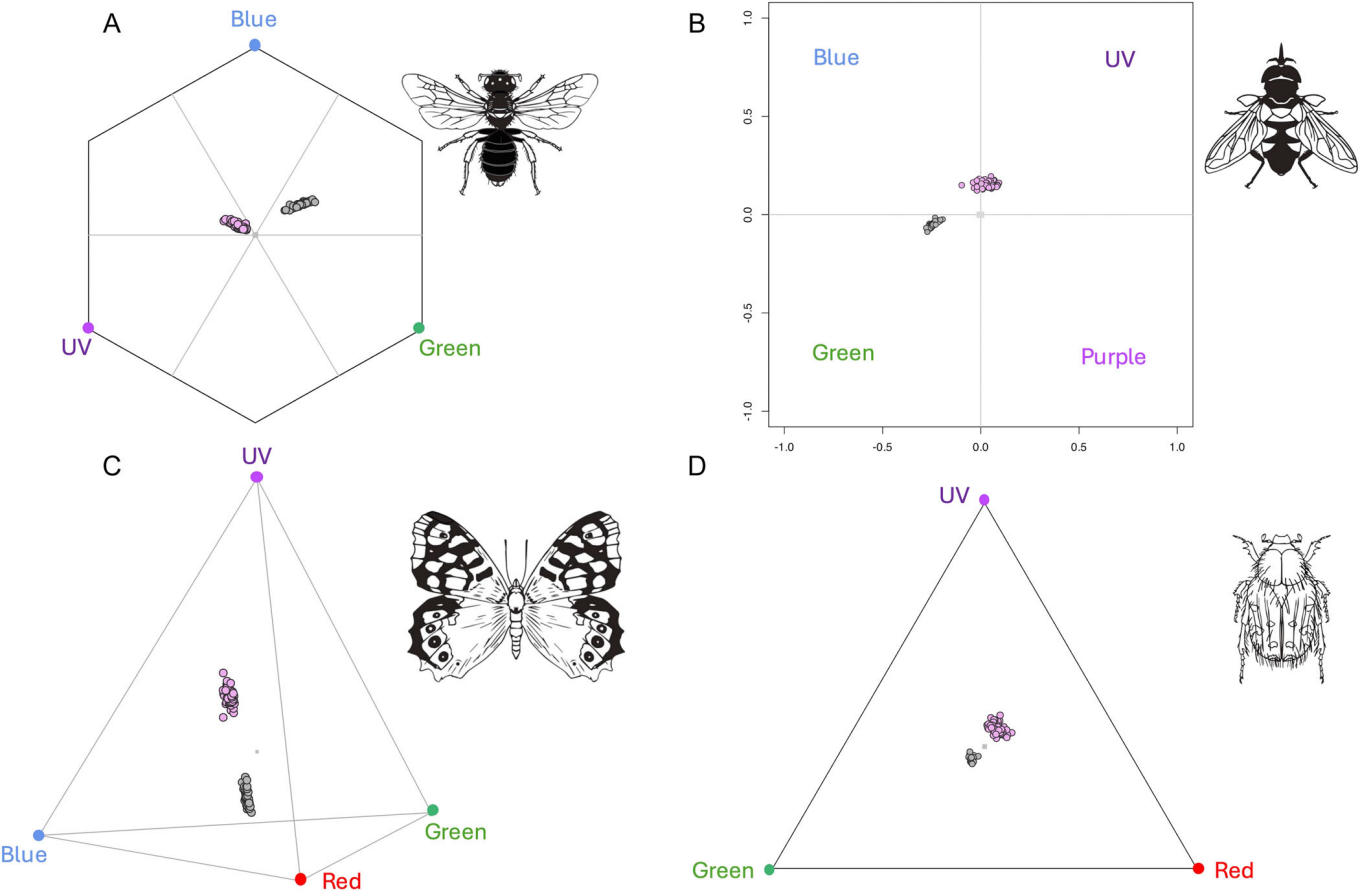
Pollinators visual system models showing the loci of spring (pink points) and summer (grey points) flowers. (A) hexagonal color space for the trichromatic vision of *Apis mellifera* (Apidae, Hymenoptera). (B) Categorical color model for *Eristalis tenax* (Syrphidae, Diptera). (C) Tetrahedron model for *Papilio xuthus* (Papilionidae, Lepidoptera). (D) Maxwell triangle for *Pygopleurus israelitus* (Glaphyridae, Coleoptera). To calculate the color loci, we used background spectra corresponding to each season (i.e. green leaves for spring and soil for summer; see Methods). The colored circles on the vertices represent the maximum signals in the blue, green, UV and red photoreceptors. Insect silhouettes from Divulgare (divulgare.net) under a Creative Commons license (http://creativecommons.org/licenses/by-nc-sa/3.0).

Spring and summer flowers occupied distinct regions within the color vision models for each of the four main functional pollinator groups. In the color hexagon of hymenopterans, spring flowers occurred in the UV‐blue region of the color hexagon, while summer flowers were located in the blue‐green region (Figure 2A). In the categorical color vision model for dipterans, spring flowers were positioned in the blue and UV regions, while summer flowers tended to appear in the green region (Figure 2B). In the tetrahedral color space for lepidopterans, spring flowers were positioned between the achromatic center and the UV vertex, with summer flowers shifting toward the red vertex (Figure 2C). Finally, in the triangle color model for coleopterans, spring flowers were mostly placed in the UV sector, and summer flowers appeared in the green sector (Figure 2D).

**Figure 3:**
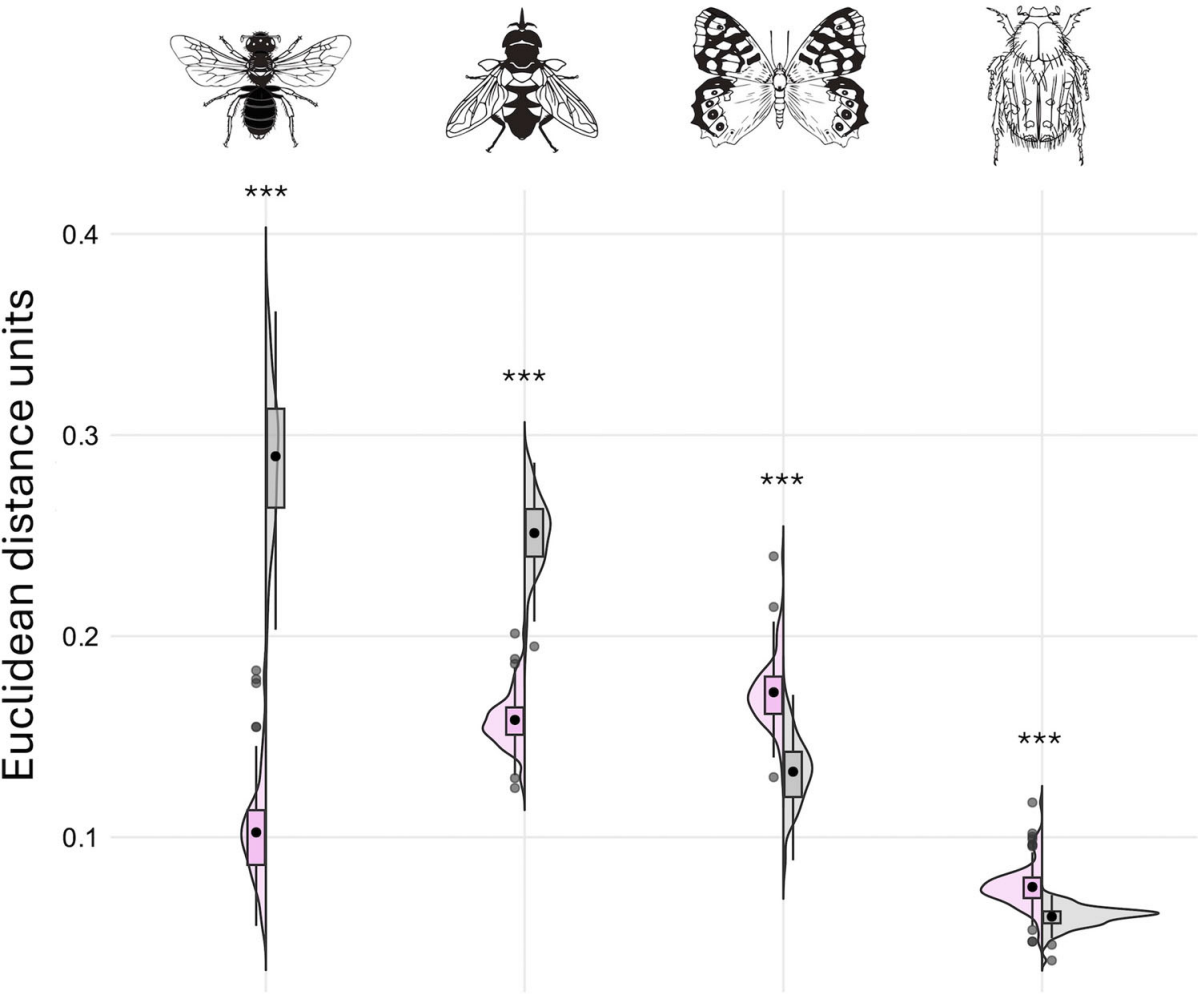
Violins with boxplots representing the distribution of chromatic contrast values (Euclidean distance units) of spring and summer flowers (pink and grey areas, respectively). Values were obtained from the vision models of *Apis mellifera* (Apidae, Hymenoptera), *Eristalis tenax* (Syrphidae, Diptera), *Papilio xuthus* (Papilionidae, Lepidoptera), and *Pygopleurus israelitus* (Glaphyridae, Coleoptera). To calculate the color loci, we used background spectra corresponding to each season (i.e. green leaves for spring and soil for summer; see Methods). Points represent outliers. Asterisks represent statistical differences between spring and summer flowers after linear mixed models analysis for repeated measures. ***P < 0.0001. Insect silhouettes from Divulgare (divulgare.net) under a Creative Commons license (http://creativecommons.org/licenses/by-nc-sa/3.0).

Spring and summer flowers also differed in chromatic contrast, with distinct patterns across pollinator groups (Figure 3). Summer flowers showed significantly higher chromatic contrast values than spring flowers in both the hymenopteran (mean ± SE: 0.289 ± 0.004 EU vs. 0.102 ± 0.003 EU; *t* = 40.02, *P* < 0.0001) and dipteran color spaces (0.251 ± 0.002 EU vs. 0.158 ± 0.001 EU; *t* = −38.03, *P* < 0.0001). Conversely, spring flowers displayed significantly higher chromatic contrast values than summer flowers in both the lepidopteran (0.172 ± 0.002 EU vs. 0.133 ± 0.002 EU; *t* = −15.32, *P* < 0.0001) and coleopteran color spaces (0.075 ± 0.001 EU vs. 0.060 ± 0.001 EU; *t* = −10.90, *P* < 0.0001). Regarding achromatic contrast in the hymenopteran vision model, summer flowers exhibited significantly lower values compared to spring flowers (mean ± SE: 0.175 ± 0.004 EU and 0.310 ± 0.004 EU, respectively; *t* = −25.70, *P* < 0.0001; Appendix S2).

When comparing the chromatic contrast of spring and summer flowers against their own seasonal backgrounds versus the background of the opposite season (i.e., spring flowers on a summer background and vice versa), distinct patterns emerged across pollinator visual systems. Spring flowers exhibited significantly higher chromatic contrast against their own seasonal background in the visual systems of flies and butterflies, whereas the opposite pattern was observed in the bee visual system. Summer flowers displayed significantly higher chromatic contrast against their own seasonal background across all visual systems, except for butterflies (Appendix S3). Additionally, the achromatic contrast perceived by bees was higher using the spring background in both spring and summer flowers.

### Reflectance spectra simulations and spontaneous anthocyanin‐lacking mutants

**Figure 4:**
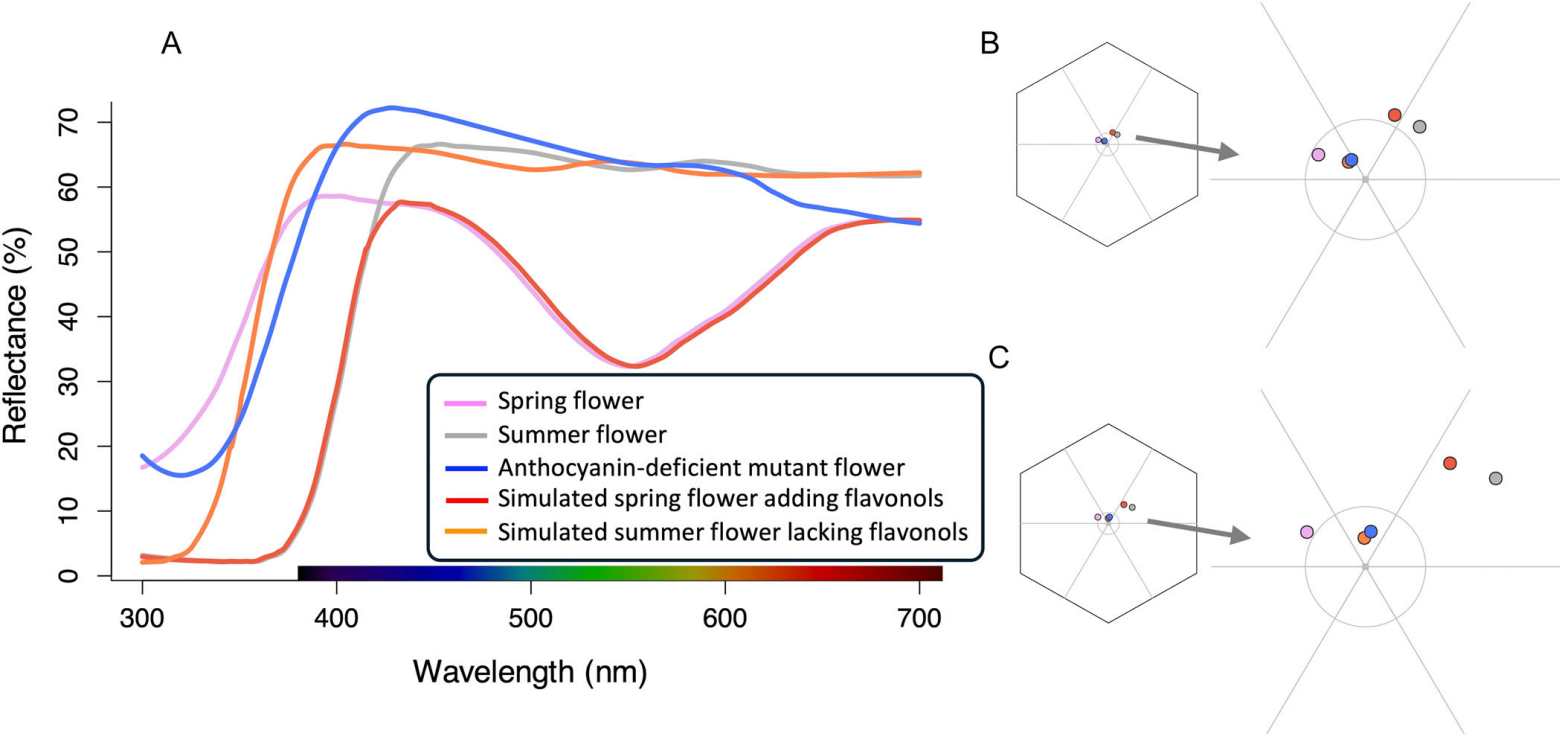
Reflectance spectra (A) and their representation in the bee hexagon color space using spring background (B) and summer background (C) for simulated spectrum of spring flowers adding flavonols, simulated spectrum of summer flowers lacking flavonols, mean spectra of spring flowers from spontaneous anthocyanin‐deficient mutants, and mean spectra of spring and summer flowers of Negratín population. In the hexagon, grey circle represents the discriminability threshold for honeybees under absolute conditioning set at 0.11 EU (Dyer et al., 2012).

In the bee visual model, the simulated reflectance spectrum of spring flowers adding flavonols showed a chromatic contrast of 0.129 EU against the spring background and 0.244 EU against the summer background (Figure 4). These values approached the mean chromatic contrast of summer flowers (0.144 EU and 0.288 EU, respectively). In contrast, the simulated spectrum of summer flowers lacking flavonols produced a chromatic contrast of just 0.045 EU against the spring background and 0.053 EU against the summer background, well below the discrimination threshold (0.11 EU) and less than half the mean value observed in spring flowers (0.102 EU and 0.124 EU, respectively). Notably, mean reflectance of white spring flowers from spontaneous anthocyanin‐deficient mutants (presumably also lacking flavonols) exhibited a chromatic contrast of 0.045 EU and 0.065 EU, respectively, with color loci closely matching those of the simulated flavonol‐lacking summer flowers (Figure 4).

### Absorbance spectra of spring and summer flowers

Spring flowers showed a distinct absorbance peak in the green region characteristic of anthocyanin pigments, while this peak was absent in summer flowers (Figure 1B). Both spring and summer flowers displayed absorbance peaks in the UV region, associated with the presence of UV‐absorbing flavonoids. However, this peak was broader and more intense in summer flowers. Consequently, spring flowers produced 5.32 ± 1.76 mg/g FW (mean ± SE) of total anthocyanins (cyanidin‐3‐glucoside equivalents), whereas summer flowers produced only trace amounts (0.28 ± 0.59 mg/g FW). In contrast, UV‐absorbing flavonoid content was 2.5 times higher in summer flowers than in spring flowers (82.59 ± 4.23 vs. 33.62 ± 1.10 mg/g FW; kaempferol‐3‐glucoside equivalents).

### Phenolic composition of spring and summer flowers

UHPLC analysis of methanolic petal extracts from summer flowers identified 20 phenolic compounds (excluding anthocyanins; see Materials and Methods): 11 flavonoids—including six flavonols, one flavone, one isoflavone, one chalcone, and one dihydroflavonol—and nine non‐flavonoid phenolics from upstream biosynthetic pathways, comprising three hydroxycinnamic acids, two coumarins, two phenolic aldehydes, and two hydroxybenzoic acids (Table 1). Spring flowers contained only 10 of these phenolic compounds, with flavonoids limited to the flavonols kaempferol and kaempferol‐3‐O‐glucoside. The remaining non‐flavonoid phenolics matched those of summer flowers, except for the absence of the coumarin umbelliferone.

**Table 1:** UHPLC‐HRMS/MS analysis of phenolic compounds (excluding anthocyanins) detected in petal methanol extracts of lilac spring flowers and white summer flowers of *Moricandia arvensis*.

						Spring flowers	Summer flowers	
Chemical family/compound	RT (min)	[M−H]− (*m/z*)	Identification method	Δ *m/z* (ppm)	Δ RT (sec)	Peak area (×10^6^ g^−1^)	Peak area (×10^6^ g^−1^)	t‐test (p*‐*value)
**Flavonols**								
Myricetin	4.24	317.03018	IP, FI	−0.5336	−1.7517	0.00	0.78	‐
Quercetin	4.91	301.03546	IP, FI	0.2705	−1.7325	0.00	2.46	‐
Isorhamnetin	5.59	315.05093	IP, FI	−0.3052	−1.7890	0.00	1.68	‐
Quercetin‐3‐O‐glucoside	5.98	463.08844	RT, IP, FI	0.4522	−0.0253	0.00	1.60	‐
Kaempferol‐3‐O‐glucoside	6.07	447.09384	RT, IP, FI	0.6417	−0.0284	1.60	37.90	0.0009
Kaempferol	7.17	285.04050	RT, IP, FI	0.1292	−0.0206	0.91	41.31	0.009
**Flavones**								
Apigenin‐7‐O‐glucoside	6.01	431.09811	IP, FI	−0.3106	1.0202	0.00	0.39	‐
Luteolin 7‐O‐glucuronide	6.23	461.07047	PI	−4.3895	0.1665	0.00	1.31	‐
**Isoflavones**								
Daidzin	5.43	415.10355	IP	0.2075	0.3447	0.00	0.23	‐
**Chalcones**								
Marein	5.26	449.10922	IP	0.6511	0.1111	0.00	0.10	‐
**Dihydroflavonols**								
Dihydrokaempferol	6.53	287.05634	RT, IP, FI	0.7854	−0.0070	0.00	0.32	‐
**Hydroxycinnamic acids**								
Caffeic acid	3.99	179.03500	RT, IP, FI	0.1322	−0.0579	1.02	2.16	0.021
Sinapic acid	5.23	223.06126	IP, FI	−0.1234	0.3631	0.29	0.78	0.402
Ferulic acid	5.87	193.05054	IP, FI	−0.4812	0.6968	133.39	315.80	0.003
**Coumarins**								
Aesculetin	3.90	177.01933	RT, IP, FI	−0.1561	−0.0439	0.35	1.09	0.006
Umbelliferone	5.47	161.02448	RT, IP	0.3422	0.9775	0.00	0.33	‐
**Phenolic aldehydes**								
Vanillin	4.52	151.04002	IP, FI	−0.6089	1.1141	0.06	0.76	0.019
Isovanillic acid	4.77	167.03502	IP, FI	−0.1324	0.4603	6.94	8.11	0.502
**Benzoic and Hydroxybenzoic acids**								
p‐hydroxybenzoic acid	3.09	137.02438	IP, FI	−0.3774	−0.2947	0.73	2.20	0.026
Benzoic acid	3.85	121.02943	IP	−0.4176	−1.8007	0.36	0.39	0.856

Identification was conducted with comparison with standards based on retention time (RT), isotopic pattern (IP), and exact masses of fragment ions (FI) or pseudomolecular ions (PI). Δ (Delta) represents the error between experimental and theoretical values. Peak intensity area was calculated as the mean value from seven plants per flower type. Results of the paired t‐tests comparing compound peak areas between spring and summer flowers are presented.

**Figure 5:**
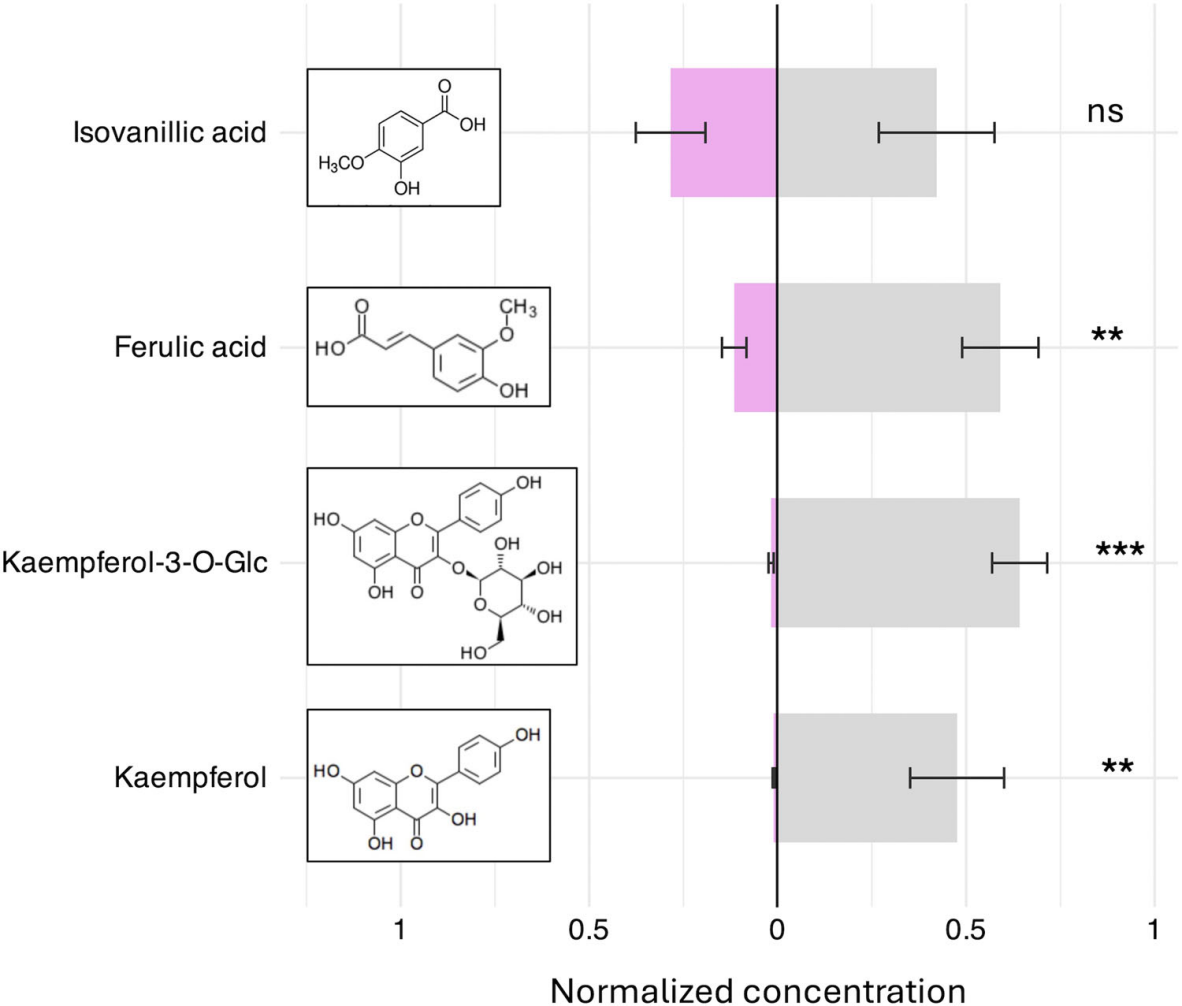
Comparison of the concentration of the four more abundant phenolic compounds between spring and summer flowers (lilac and grey bars, respectively). Mean and S.E. of the normalized concentration (intensity peak area g^−1^) obtained in the UHPLC‐ESI‐MS/MS analysis. Chemical structure of each compound is depicted. The results of the paired t‐test (n = 7), comparing each compound concentration between spring and summer flowers, are reported. ns, not significant; **P < 0.01; ***P < 0.001.

Summer flowers exhibited generally higher concentrations of phenolic compounds, as indicated by relative peak intensities (Table 1; Appendix S4). Thus, seven of the 10 compounds shared by both flower types exhibited significantly higher mean concentrations in summer flowers. Focusing on the phenolic compounds with higher relative concentrations (mean intensity peak area/g > 5), only isovanillic acid did not differ significantly between flower types (Table 1; Figure 5). Ferulic acid exhibited the highest relative intensity in both types of flowers, with summer flowers showing a normalized concentration 4.4 times higher than that of spring flowers (Figure 5). The flavonols kaempferol and kaempferol‐3‐O‐glucoside were present at relatively high intensity in summer flowers but exhibited very low intensity in spring flowers (Table 1), with normalized concentration differences of 45.2‐fold and 36.8‐fold, respectively; Figure 5).

## DISCUSSION

### Seasonal floral color shift preserves conspicuousness

This study demonstrates that the white summer flowers of *Moricandia arvensis* are not simply lilac spring flowers that have lost anthocyanins. Rather, summer flowers exhibit elevated levels of shared phenolic compounds and the biosynthesis of novel metabolites that are absent in spring flowers. Notably, kaempferol and kaempferol‐3‐O‐glycoside were exclusively detected in the summer phenotype. These phenolic compounds exhibit broad absorption across the UV spectrum, with absorption maxima at 364 nm and 350 nm, respectively (Mabry et al., 1970; Harborne and Williams, 2000). The significantly higher concentrations of these compounds in summer flowers likely explain the absence of floral reflectance in the 350–400 nm range, which contrasts with the UV reflectance pattern observed in spring flowers. In addition, summer flowers produced low amounts of quercetin and quercetin‐3‐O‐glycoside, both of which exhibit strong absorption within the UV spectral region (Harborne and Williams, 2000; Agati and Tattini, 2010), potentially contributing to their distinctive UV reflectance profile. In contrast, ferulic acid demonstrates strong UV absorption within the 260–350 nm range, with an absorption maximum at 322 nm (Anouar et al., 2009; Wang et al., 2022). Its high accumulation in both floral phenotypes suggests that it is the primary compound responsible for the UV absorption observed between 300 and 350 nm, a characteristic shared by both spring and summer flowers. Isovanillic acid was also detected in both phenotypes, although at considerably lower concentrations. Given that it predominantly absorbs at wavelengths below 300 nm (Robbins, 2003), its contribution to UV absorption within the analyzed range is likely negligible.

The loss of anthocyanins and the resulting shift in floral coloration may reduce floral detectability by certain pollinators. This effect was evident in visual models for butterflies and coleopterans, where summer flowers exhibited reduced chromatic contrast relative to spring flowers. A similar decline was observed in the achromatic contrast perceived by hymenopterans. In contrast, chromatic contrast values for hymenopterans and dipterans displayed an opposing pattern. For hymenopterans—the most effective pollinators during both spring and summer (Gómez et al., 2020, 2024, 2025a)—the chromatic contrast of both floral phenotypes approached or exceeded the discrimination threshold under absolute conditioning (Dyer et al., 2012), suggesting that these flowers remain perceptually salient to bees despite changes in pigment composition. Indeed, an experiment offering both flower types simultaneously demonstrated that bees visited both spring and summer flowers (Gómez et al., 2020, 2025a). In these experiments, however, spring flowers received a higher visitation rate than summer flowers (Gómez et al., 2020, 2025a), a finding that may seem incongruent with the much higher chromatic contrast of summer flowers reported in the present study. This apparent discrepancy highlights that increased floral conspicuousness does not necessarily correlate with increased pollinator visitation (Garcia et al., 2017). Other floral attributes—such as shape, size, scent, and the availability of rewards like pollen and nectar—also significantly influence pollinator behavior (Schiestl and Johnson, 2013; Gómez et al., 2015; Rusman et al., 2019). It is important to acknowledge that visual models are theoretical constructs; thus, their ecological relevance should be validated through psychophysical studies and behavioral assays conducted under natural conditions (Renoult et al., 2017; van der Kooi and Kelber, 2022; Lunau and Dyer, 2024). On the other hand, our findings show that background influences the conspicuousness of flower color to pollinators, aligning with previous studies comparing green foliage and bare soil (Bukovac et al., 2017; Finnell and Koski, 2021). However, the comparisons of achromatic and chromatic contrasts between flower color morphs in their native *versus* opposite seasonal backgrounds did not reveal a consistent pattern across pollinator visual models.

The number and position of inflection points in the reflectance curve (i.e., wavelengths with maximum slope) are critical for color discrimination by bees (Chittka and Waser, 1997; Shrestha et al., 2013). The bee visual system exhibits peak sensitivity around 400 and 500 nm (Chittka and Menzel, 1992), and floral signals with inflection points near these wavelengths are typically well discriminated (Chittka and Menzel, 1992; Dyer et al., 2012; Rodríguez‐Sambruno et al., 2024). The lilac spring flowers of *Moricandia arvensis* exhibited an inflection point near 350 nm—outside the primary sensitivity range—but also a second inflection point around 500 nm (Figure 1A). In contrast, the white summer flowers showed a single inflection point located near 400 nm. Most white flowers display reflectance spectra with marker points near 400 nm (Chittka et al., 1994; Reverte et al., 2016; del Valle and Narbona, unpublished results), a pattern likely shaped by selective pressures to suppress UV reflectance. In such flowers, elevated UV reflectance would significantly reduce visual conspicuousness and potentially impair pollinator attraction and reproductive success (Kevan et al., 1996; Lunau et al., 2011; del Valle et al., 2025). Thus, the spectral characteristics of both floral phenotypes of *M. arvensis* further suggest that they can be effectively distinguished by hymenopteran visual systems (Chittka, 1992; Shrestha et al., 2013; Rodríguez‐Sambruno et al., 2024).

On the other hand, simulated reflectance spectra of white flowers lacking both flavonols and anthocyanins exhibited very low chromatic contrast values, making them poorly distinguishable against both spring and summer backgrounds in bee visual system (Chittka and Waser, 1997; Kevan et al., 2001). The reflectance profiles and color loci location in bee visual model of these simulated flowers closely matched those observed in anthocyanin‐deficient mutants. These findings underscore the critical role of UV‐absorbing phenolic compounds in enhancing the visual detectability of white flowers to bee pollinators. In contrast, the addition of flavonols to the simulated reflectance spectrum of spring flowers resulted in a chromatic contrast comparable to that of summer flowers in both spring and summer background. This suggest that the presence of both flavonoid groups, anthocyanins and flavonols, does not increase conspicuity compared to flowers producing only flavonols.

### Differences in phenolic compound composition and its role on heat protection

Summer flowers of *M. arvensis* produced twice as many phenolic compounds as spring flowers and accumulated them at significantly higher concentrations. Ferulic acid, the dominant compound in both flower types, was 4.4 times more concentrated in summer flowers. This compound is synthesized in the early stages of the general phenylpropanoid pathway and derives from caffeic acid, which was also present in summer flowers (Strack, 1997; Khan et al., 2024). Ferulic acid is widely recognized as a secondary metabolite produced in response to abiotic and biotic stresses, due to its exceptional antioxidant properties (Khan et al., 2024). This compound catalyzes the formation of stable phenoxy radicals, which protect plant cells from oxidative damage induced by UV radiation, drought, or salt stress (Sharma et al., 2019; Khan et al., 2024), with particularly strong protective effects under heat stress conditions (Cheng et al., 2018; Rehman et al., 2024). Although primarily studied for its protective role in photosynthetic tissues, high concentrations of ferulic acid have also been reported in the petals of various plant species (Xiong et al., 2014; Juárez‐Trujillo et al., 2018, Chamani et al., 2020; Han et al., 2023). Therefore, the marked accumulation of ferulic acid in summer flowers of *M. arvensis* may contribute to enhanced floral resilience under the torrid temperatures of the summer in the Mediterranean Basin.

Kaempferol and quercetin, along with their glycosylated derivatives, identified in the summer flowers of *Moricandia arvensis*, are among the most frequently occurring flavonoids in plants and their floral tissues (Winkel‐Shirley, 2001; Iwashina 2015; Isnaini et al., 2018). They are considered secondary metabolites that plants induce in response to environmental stresses, including high temperatures (Ryan et al., 2001; Yang et al., 2018; Borghi et al., 2019; Xiao et al., 2021). Due to their antioxidant properties, these flavonols help detoxify the harmful H₂O₂ molecules produced under stress (Jan et al., 2021). Summer flowers accumulated 3‐O‐glycosylated derivatives of both kaempferol and quercetin, which generally exhibit lower free radical scavenging activity compared to their aglycones (Xiao et al., 2021). However, recent studies have shown that flavonoid glycosylation may play a crucial role in the plant's response to high‐temperature stress (Behr et al., 2020; Dong et al., 2025; Zhao et al., 2025). Using rice mutant lines for overexpression and knockout of *OsDUGT1*, a glycosyltransferase gene, Dong et al., (2025) demonstrated that this gene is involved not only in the glycosylation of kaempferol and quercetin, but also in the broader regulation of flavonoid metabolism via glycosylation. This gene, along with a MYB transcription factor involved in its activation, also promotes the expression of heat stress‐related genes, including those involved in temperature regulation, membrane transport, and active oxygen scavenging (Dong et al., 2025; Zhao et al., 2025). In *M. arvensis*, a transcriptomic analysis comparing the flavonoid biosynthetic pathways in spring and summer flowers, revealed differential expression of several structural and regulatory genes, including flavonoid‐3‐O‐glucosyltransferase and a MYB transcription factor (Gómez et al., 2020). Taken together, these findings suggest that the elevated accumulation of flavonols and ferulic acid in summer flowers may represent a coordinated floral response to high summer temperatures.

Anthocyanins are particularly vulnerable to high temperatures, which promote their degradation and lead to a reduction in color intensity or complete fading (Castañeda‐Ovando et al., 2009; Zhao et al., 2021). Reproductive tissues of plants are especially susceptible to heat stress (Resentini et al., 2023). In flowers, temperatures exceeding 30°C inhibit anthocyanin biosynthesis and activate degradative pathways (Nozaki et al., 2006; Zhao et al., 2021), although the underlying regulatory mechanisms remain largely unresolved (Wang et al., 2024). *Moricandia arvensis* exhibits an extended blooming period, spanning from late winter to late summer, and the production of summer flowers is a plastic and reversible response to elevated temperatures (Gómez et al., 2024). The petals at the early anthesis stage of summer flowers are completely white (Narbona E., personal observations), suggesting that elevated summer temperatures effectively suppress anthocyanin biosynthesis. Our data show an increase in the number and concentration of phenolic compounds in these flowers, suggesting a redirection of anthocyanin biosynthesis towards the production of UV‐absorbing flavonoids and other phenolic compounds, as observed in other species (Wheeler et al., 2023). Notably, a previous study on selective agents influencing floral plasticity in *M. arvensis* found that selection favored lower kaempferol content in both spring and summer flowers, but this outcome likely reflects the non‐adaptive nature of floral plasticity in this species (Gómez et al., 2024). Although the elevated accumulation of phenolic compounds in summer flowers may protect petals from high temperatures, our findings suggest an additional key role in preserving floral conspicuousness to bees and other pollinator groups.

## CONCLUSIONS

We demonstrate here that the white summer flowers of *Moricandia arvensis* are not simply anthocyanin‐deficient variants of the lilac spring flowers. Summer flowers not only exhibit higher concentrations of compounds present in spring flowers, particularly flavonol derivatives, but even synthesize novel phenolic compounds. This seasonal variation in phenolic composition, driven by elevated summer temperatures, altered floral reflectance and absorbance spectra, influencing flower perception by different pollinator groups. It is noteworthy that these biochemical and functional changes occur within the same individual, highlighting that this response is phenotypically plastic. The increased production of phenolic compounds, particularly flavonoids, in flowers exposed to heat stress has been previously documented (Muhlemann et al., 2018; Borghi et al., 2019; Rehman et al., 2024). In this study, we underline the critical role of UV‐absorbing phenolic compounds in the production of summer white flowers, compensating for the loss of anthocyanins and making them easily distinguishable from their background to key pollinator groups. These findings illustrate how a response to an environmental stressor can influence how the flower is perceived by pollinators, highlighting the complex ecological interactions that drive the evolution of multifunctional traits.

## AUTHOR CONTRIBUTIONS

E.N., F.P., C.A., A.G.‐M., L.N. and J.M.G. conceived the study and conducted fieldwork. J.D. and E.N. performed pollinator visual modelling. All authors wrote the paper and contributed to revisions.

## CONFLICT OF INTEREST STATEMENT

Eduardo Narbona is Special Issue Editor for the *American Journal of Botany* but took no part in the peer‐review and decision‐making processes for this paper.

## Supporting information


**Appendix S1**. UV‐visible reflectance spectra of spring and summer flowers of *Moricandia arvensis* from Negratín population (pink and grey lines, respectively).

**Appendix S2**. Violins with boxplots representing the distribution of achromatic contrast values (Euclidean distance units) obtained from the vision models of *Apis mellifera* (Apidae, Hymenoptera).

**Appendix S3.** Comparisons of the chromatic contrast of spring and summer flowers against their native backgrounds versus the opposite seasonal background in the visual system of bees, flies, butterflies and beetles. Achromatic contrast was only calculated for bees.

**Appendix S4.** Peak intensity area (x10^6^ g‐1) of each phenolic compound obtained from the UHPLC‐HRMS/MS analysis for spring and summer flowers.

## Data Availability

Reflectance spectra and absorbance spectra of methanol extracts are available online via RIO Digital Repository at https://hdl.handle.net/10433/24297.
