# Supplementary material for: Heat drastically alters floral color and pigment composition without affecting flower conspicuousness
Source: Am J Bot. 2025 Sep 9;113(1):e70096. doi: 10.1002/ajb2.70096 (PMC12816441; doi:10.1002/ajb2.70096)

Appendix S1. UV-visible reflectance spectra of spring (pink) and summer (grey) flowers of *Moricandia arvensis* from the Negratín population.

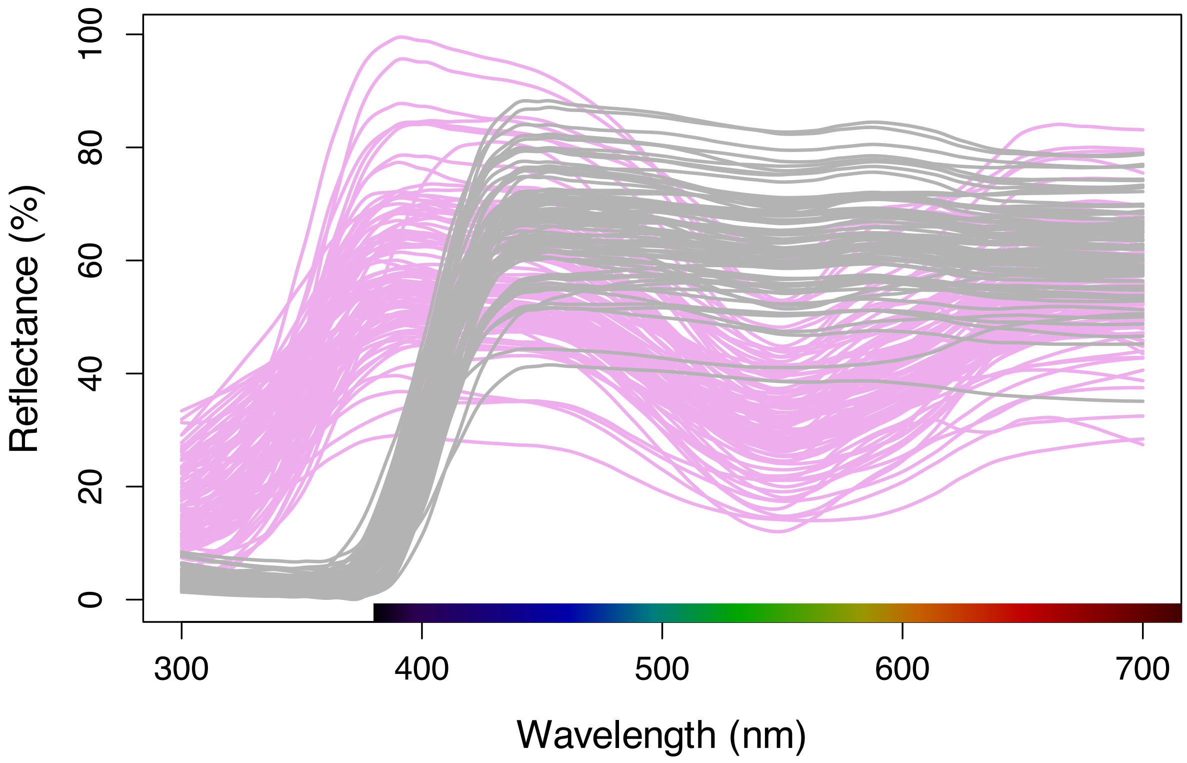

Supplement: Supplementary file 1 — Appendix S1. UV‐visible reflectance spectra of spring and summer flowers of Moricandia arvensis from Negratín population (pink and grey lines, respectively). [file AJB2-113-e70096-s004.pdf]
