# Supplementary material for: Heat drastically alters floral color and pigment composition without affecting flower conspicuousness
Source: Am J Bot. 2025 Sep 9;113(1):e70096. doi: 10.1002/ajb2.70096 (PMC12816441; doi:10.1002/ajb2.70096)

Appendix S2. Violin plots with box plots representing the distribution of achromatic contrast values (Euclidean distance units) of spring (pink) and summer (grey) flowers. Values were obtained from the vision model of *Apis mellifera* (Apidae, Hymenoptera). Points represent outliers. Asterisks represent significant differences between spring and summer flowers in a linear mixed model analysis for repeated measures; ***$P < 0.0001$.

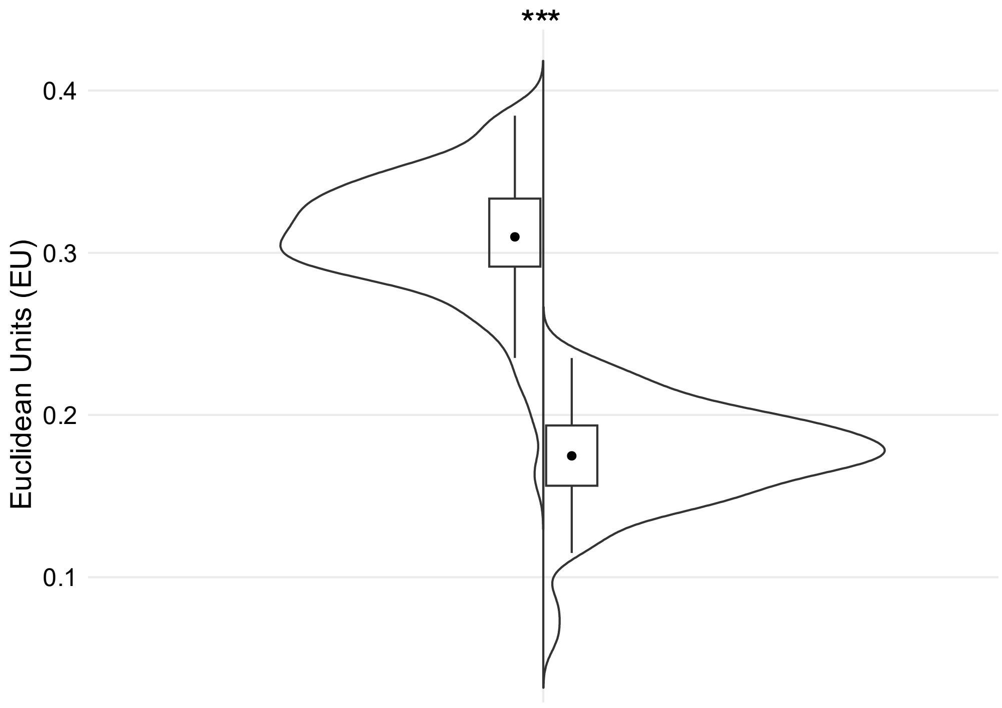

Supplement: Supplementary file 2 — Appendix S2. Violins with boxplots representing the distribution of achromatic contrast values (Euclidean distance units) obtained from the vision models of Apis mellifera (Apidae, Hymenoptera). [file AJB2-113-e70096-s001.pdf]
